# Inequality atop excess: a nonlinear decomposition of wealth-related inequality in caesarean section delivery in Punjab, Pakistan

**DOI:** 10.3389/fpubh.2026.1930664

**Published:** 2026-09-30

**Authors:** Muhammad Nadeem, Shahid Adil, Adel Bashatah, Mohamed N. Al Arifi, Wajid Syed, Ayesha Iqbal

**Affiliations:** 1 Department of Economics, Division of Management and Administrative Sciences, University of Education, Lahore, Pakistan; 2 Punjab Resource Improvement and Digital Effectiveness (PRIDE) Project, Planning and Development Board, Government of the Punjab, Lahore, Pakistan; 3 Department of Nursing Administration and Education, College of Nursing, King Saud University, Riyadh, Saudi Arabia; 4 Department of Clinical Pharmacy, College of Pharmacy, King Saud University, Riyadh, Saudi Arabia; 5 Division of Pharmacy Practice and Policy, School of Pharmacy, University of Nottingham, Nottingham, United Kingdom; 6 Office of Lifelong Learning and the Physician Learning Program, Faculty of Medicine and Dentistry, University of Alberta, Edmonton, AB, Canada

**Keywords:** caesarean section, concentration index, maternal health services, Oaxaca– Blinder decomposition, Pakistan, socioeconomic inequality

## Abstract

**Background:**

Caesarean section (CS) rates are rising rapidly across South Asia, and Pakistan combines over-use among advantaged groups with historically limited access among the poor. Quantifying and decomposing wealth-related inequality in CS delivery is essential for equitable obstetric policy. Decomposed by the Wagstaff–van Doorslaer method; the rich–poor gap was decomposed using a two-fold nonlinear (Yun) Oaxaca–Blinder decomposition. All estimates applied survey weights with PSU-cluster-robust inference; analyses used Python 3.12 (statsmodels 0.14). Because service-use variables lie on the causal pathway between wealth and CS, hierarchical models are reported and all decomposition results are descriptive, not causal.

**Methods:**

A cross-sectional analysis of 6,832 ever-married women with recorded last pregnancy delivery from the Punjab Health and Population Survey (PHPS) 2024–25 was done. The aim was to measure inequality by wealth in the rates of cesarean section (CS) delivery and identify the channels responsible for the inequality. The dependent variable CS delivery was binary, with the primary explanatory variable being a principal-component analysis (PCA) based household wealth index, with maternal, service use and clinical covariates. Inequality was measured using concentration curves, standard concentration index, Wagstaff-normalised concentration index, Erreygers concentration index and decomposed by Wagstaff–van Doorslaer method. The rich–poor gap was decomposed by a two-fold nonlinear (Yun) Oaxaca–Blinder decomposition. Survey weights were employed in all estimates, which were derived using PSU-cluster-robust inference; analyses were conducted in Python 3.12 (statsmodels 0.14). Because service-use variables lie on the causal pathway between wealth and CS, hierarchical models are reported and all decomposition results are descriptive, not causal.

**Results:**

The weighted CS rate was 43.3% (21.0% poorest to 64.6% richest quintile). CS delivery was concentrated among the wealthy (standard CI = 0.195, 95% CI 0.173–0.215; Erreygers = 0.337, 95% CI 0.301–0.373). In the fully adjusted model the richest quintile (OR 2.07, 95% CI 1.50–2.85), private-facility delivery (OR 3.50, 2.90–4.23) and lady-doctor attendance (OR 13.10, 9.65–17.80) showed the strongest associations. There was no multicollinearity (all VIF < 2.45) and model discrimination was good (AUC 0.824). Excluding proximate service-use variables, observed characteristics accounted for 66.9% of the 28.4-percentage-point rich–poor gap; adding service use raised this to 87.3%, with lady-doctor attendance (44.7%) and private-facility delivery (20.9%) the largest components.

**Conclusion:**

Wealth-related inequality in caesarean delivery in Punjab is predominantly associated with differential access to private, physician-led obstetric care. These associations are descriptive and cannot establish causation. Policy should pair clinical audit of elective CS indications in the private sector with financial protection for medically necessary CS among poorer women.

## 1 Introduction

Safe childbirth is a cornerstone of the international development architecture. The 2030 Agenda for Sustainable Development includes two targets related to maternal health: target 3.1 which stipulates that the global maternal mortality ratio (MMR) must be reduced to less than 70 deaths per 100,000 live births and target 3.8 which says that the member states will ensure universal health coverage (UHC), including financial risk protection and access to quality essential services (1). These targets continue an unfinished agenda of the Millennium Development Goals, which achieved a significant reduction of around 44% in the rate of global maternal mortality between 1990 and 2015, but which saw a disproportionate share of maternal deaths concentrated in South Asia and sub-Saharan Africa (2). Access to emergency obstetric care, including a caesarean section (CS) is essential for both targets. However, caesarean delivery is also the most widely used major surgery in the world, and has spread far beyond its clinical need. Global CS rates rose from about 7% of births in 1990 to 21% by 2018 and are projected to approach 29% by 2030 (3). Several times, WHO has reported that no further reductions in maternal or newborn mortality occur when rates surpass 10–15% of births delivered in health facilities (4, 5). The Lancet 2018 caesarean series revealed the remarkable co-existence of over-use and under-use: in some 60% of countries, the number of C-section deliveries was above the reference range, whereas in 25% it was below, often within the same country and across socioeconomic levels (6). The additional risks of unnecessary caesarean section are haemorrhage, infection, complications of the placenta during future pregnancies and the impact on the baby’s health (7). They are also expensive: WHO background work suggested that the world’s cost of having medically unnecessary sections is around US$2.3 billion a year, money that could be used to address the unmet need many times over (8). In terms of economic theory, caesarean delivery is a near textbook example of a failure of the market in medical care. Health care markets have been recognized as having a high degree of information asymmetry between providers and patients, uncertainty, and insurance-induced distortions of demand since Arrow’s basic analysis (9). In the Grossman model, households have an inverse demand for health and an inverse demand for medical care, as income and education are important factors of production, and expect a positive income gradient in the use of more sophisticated obstetric technology (10). On the supply side, two other findings from the physician-agency literature are relevant: when providers are paid more for a surgical delivery than a vaginal delivery, and when the patients cannot make their own clinical evaluation, additional caesareans will result, as Gruber and Owings (11) documented in the United States during the decline in fertility in the 1990s, and as Gruber, Kim and Mayzlin (12) showed using procedure-level fee differentials, and, crucially, as Johnson and Rehavi demonstrated, physician mothers who can make their own clinical evaluation are less likely to receive a caesarean than similar non-physician patients (13, 14). These demand- and supply-side factors reinforce each other in pluralistic health systems in which regulation is weak, and the private sector is strong, as is commonly seen in South Asia. The result from this is a large health inequality between rich and poor, often five to ten times higher in the richest quintile than in the most impoverished (15, 16). This is very pronounced in the case of Pakistan. The country (241.5 million people in 2023) (17) has an MMR of 186/100,000 live births above the SDG trajectory (18), and has seen its national caesarean rate rise from below 3% in the early 1990s to approximately 22% in 2017–18, with successive Demographic and Health Surveys indicating that the increase has been concentrated among urban, educated and wealthier women and in the private sector (19–21). The health-financing environment strengthens the equity arguments: roughly half of all health expenditure is paid out of pocket (22), in many districts most deliveries take place in private facilities, and in most countries, the cost of a caesarean is several times the cost of a normal delivery. Public sector options are limited for comprehensive care in emergency obstetrics, which means that the options available to a poor rural woman may be limited to none, whereas the options available to an affluent urban woman may be limited to a scheduled private caesarean at low time cost. Some recent demand-side interventions, including the social health insurance schemes which provide coverage for inpatient maternity services, are now starting to shift these constraints, and existing, province-specific evidence of the distribution of caesarean delivery is directly policy-relevant (23). This study focuses on Punjab, which is the most populous province of Pakistan with more than half of the country’s population, and is the fastest progressing province in terms of obstetric transition, having experienced the highest rate of increase in skilled birth attendance and institutional delivery and a provincial rate of caesarean deliveries of over 43% in the recent PHPS 2024–25 (24). It is therefore a particularly harsh test of the distribution of surgical childbirth in Punjab to determine whether it is going to those who need it or to those who can afford it. While descriptive wealth gradients in the use of caesarean section in Pakistan have been reported (19, 20, 25), there have been few attempts to formally measure levels of inequality using rank-dependent indices, or to decompose that inequality into its proximate economic sources. The Wagstaff–van Doorslaer regression-based decomposition apportions the index among determinants (26) and the Oaxaca–Blinder decomposition developed in labour economics to separate endowment from coefficient (returns) effects in group wage gaps (27–29) answers the counterfactual question of how much of the rich–poor gap would close if poorer women possessed richer women’s observable characteristics.

Despite this literature, there are three gaps. Firstly, most Pakistani evidence confirms the wealth related differences in caesarean rates but is restricted to measuring this gradient in simple terms such as ‘richer’ and ‘poorer’ without standardising the measures using rank-based indices comparable across different populations. Secondly, few studies break down that inequality into its constituent pathways, making it difficult to determine if the wealth gradient primarily acts as a poverty gradient, through the purchasing power of households, or as an inequality gradient, through differential access to specific types of provider and facilities, which, directly, may have implications for targeting policy to household incomes versus provider behaviour. Third, linear methods are generally unsuitable for a binary outcome, and most existing decomposition applications in this area are linear. This paper seeks to fill all three gaps, quantifying inequality with both standard and the Wagstaff-normalised indices, as well as the Erreygers index, decomposing the concentration index and rich–poor gap, with the latter being decomposed nonlinearly, given that it represents a binary outcome, and separates distal from proximate determinants by a hierarchical specification. Its contribution is to shift from describing the inequality as a fact to identifying pathways, quantitatively, that create the inequality.

This study reports, and also distinguishes between, decompositions with and without the proximate variables (service-use variables: place of delivery, attendance), in the spirit of Mosley and Chen (30); the distal socioeconomic factors (wealth, education, region) have a partial effect on the caesarean outcome through the proximate service-use channels. The present study measures the inequality in the use of caesarean section, identifies the adjusted correlates of that inequality and quantifies the contributions of observable characteristics to the rich/poor gap, and subsequently draws policy implications with regard to provider-payment policy, the role of the private sector in governance and financial protection under the UHC agenda in Pakistan.

## 2 Materials and methods

### 2.1 Data source and variable definitions

Analysis of data from the Punjab Health and Population Survey (PHPS) 2024–25 conducted in Punjab, Pakistan on ever-married women of reproductive age. The survey is a stratified two-stage cluster survey where the primary sampling units (PSUs) were randomly selected from the urban and rural population across the districts of Punjab, and households were sampled within PSUs, while survey weights were provided with the microdata. The woman’s questionnaire includes the household-roster, background, reproductive-history, family-planning, antenatal and delivery modules; the delivery module captures the outcome, mode of delivery, place, attendance, and timing of decision of her last pregnancy. The data for 6,847 respondents have been analyzed. Definitions and categories of all variables are given in Table 1. This is a cross-sectional study that is reported as per the STROBE checklist for cross sectional observational studies. The flow of participants is as: 26,598-woman records were in the survey file, 7,075 records had an unknown delivery module, 6,840 records had a recorded last-pregnancy delivery outcome and were eligible, and 8 records had an unknown highest completed grade, which were excluded yielding an analytical sample of 6,832. The missing data was minimum for all core variables (outcome, wealth index, 0.00%; pregnancy order, 0.00%; place of delivery, 0.03%; birth attendant, 0.12%; and antenatal care, 0.12%). A complete-case sensitivity analysis (*n* = 6,076) yielded materially identical estimates; pregnancy complications were missing for 757 women (11.07%) of which were coded as “no reported complication” in Model 3. No imputation was performed. Python 3.12.3, statsmodels 0.14.1, pandas 2.2, scikit-learn 1.4 were used for analyses. The explicit stratification in the survey was by region (urban/rural), which was included as a covariate in all models, and district fixed effects absorb the geographic dimension of the sampling frame.

**Table 1:** Variable description.

Variable	Type/measurement	Definition and categories
Dependent variable		
Caesarean section delivery	Binary	1 = caesarean section; 0 = normal or assisted vaginal delivery.
Independent variables		
Household wealth	Continuous score; weighted quintiles	Wealth index constructed via principal component analysis (PCA) from 17 binary household durable-goods and amenities indicators (electricity, radio, television, landline telephone, computer/laptop, refrigerator, gas heater, cooking range, electric iron, bed, sofa, closet, wall clock, washing machine, air cooler, microwave oven, and air conditioner), following Filmer and Pritchett (39). The first principal component (35.4% of total variance; all factor loadings positive, Appendix Table A1) represents the continuous wealth score. Weighted quintiles (Q1 = poorest to Q5 = richest) were used for descriptive and regression analyses.
Place of delivery	Categorical (5 categories)	0 = Home; 1 = Public facility (teaching, district- and tehsil-headquarters hospitals, rural health centres, basic health units, maternal and child health (MCH) centres, government dispensaries, family-welfare facilities); 2 = Private facility (private hospitals, private nurse, private MBBS clinics, and NGO clinics); 3 = Community health workers (lady health workers, community midwives, and outreach programme workers); 4 = Others (deliveries attended by nutrition assistants, vaccinators, traditional practitioners (hakeem, homeopath), pharmacies and unclassified).
Birth attendant	Binary	1 = delivery attended by a lady doctor (MBBS degree); 0 = any other attendant.
Women’s education	Categorical (5 categories)	Classified according to the Pakistani school structure: none; primary (up to class 5); middle (class 6 to 8); secondary (class 9 to 10); higher (above matriculation).
Region	Binary	Urban versus rural place of residence.
Maternal age	Continuous	Age of the mother in years.
Pregnancy order	Continuous/ordinal	All pregnancies including losses are counted; therefore, it is not parity, and the term pregnancy order is used throughout.
Timing of decisions and clinical variables		
Timing of caesarean decision	Binary	Elective (pre-labour) = caesarean decided before the onset of labour; Emergency = caesarean decided after the onset of labour.
Antenatal care (ANC)	Binary	1 = four or more antenatal contacts during the index pregnancy; 0 = fewer than four contacts (no antenatal care coded as 0).
Pregnancy complications	Binary	Report of serious health problems during the index pregnancy.

PCA, principal component analysis; MCH, maternal and child health; ANC, antenatal care; MBBS, Bachelor of Medicine, Bachelor of Surgery.

### 2.2 Statistical analysis

The analysis was carried out in five phases. First, summary statistics and bivariate CS rates were calculated and design-adjusted Wald tests for association were performed. Secondly, Spearman rank correlations were used to describe correlation between pairs of variables. Third, the private-facility delivery, lady-doctor attendance, and clinical variables (antenatal care 4 + contacts and pregnancy complications) were added in a hierarchical sequence to create Model 2 and Model 3, respectively, of the logistic regressions with PSU-clustered standard errors to estimate adjusted odds ratios (ORs). Fourth, the measurement of wealth-related inequality was based on the concentration curve and concentration indices for women ranked by the continuous household wealth score; standard errors from 500 PSU-cluster bootstrap replications are reported, as well as the standard concentration index (31, 32). The concentration index was decomposed using the Wagstaff–van Doorslaer approach (26) on the basis of weighted linear probability models, based on Models 1–3. Fifth, a two-fold Oaxaca–Blinder decomposition was used to decompose the difference in CS rates between the richest 40% and the poorest 40% of the weighted wealth distribution. A main nonlinear decomposition based on a pooled weighted logit model with averaged predicted probabilities as counterfactuals and Yun’s weights for the detailed decomposition (27–29, 33, 34) is presented; the conventional linear decomposition is also presented as a robustness check; sensitivity of decomposition to the group definition (Q5 vs. Q1; median split), to a nonlinear decomposition, and to excluding the service-use variables is reported. The results of the decomposition are interpreted throughout as descriptions of the sources of inequality measured, and not as estimates of causal effects, as the variables included as the proxies for the services used are co-determined with the caesarean decision. Results were declared to be statistically significant with a *p* value < 0.05 (two-sided).

### 2.3 Applied analytical techniques

Spearman rank correlation is descriptive as the variables are mixed binary, ordinal and skewed continuous, and it detects monotonic association and is scale-free (scale is arbitrary for the PCA score). It is important to explicitly state that pairwise correlation is not a good diagnostic for multicollinearity in a multivariable model, which is why variance inflation factors (VIF) are reported here for each regressor in each specification.

The logistic model gives estimated odds ratios via weighted maximum likelihood with PSU-cluster-robust (sandwich) inference.

Model performance is assessed by discrimination (area under the receiver operating characteristic curve, AUC), overall fit (McFadden pseudo-R^2^, AIC), accuracy (Brier score), calibration (Hosmer–Lemeshow test with ten risk deciles and the calibration slope of the outcome on the linear predictor), and influence (Cook’s distance).

The concentration index is calculated as C = (2/μ) × cov(y, R) where R is a weighted fractional rank with the outcome being binary; C, the Wagstaff-normalised index, W = C/(1 − μ), and the Erreygers index, E = 4μC, are reported, with the last index having the mirror property between attaining and falling short. The Wagstaff–van Doorslaer identity C = Σk ηkCk + GCε/μ decomposes the index into determinant contributions (elasticity × determinant concentration index) and a residual.

The Oaxaca–Blinder decomposition is implemented in its nonlinear form (33, 34): with F the logistic cdf, and β* the pooled logit coefficients, Δx = Ēr[F(x′β*)] − Ēp[F(x′β*)] allocates Δx across the determinants, while the remainder of the gap allocates the coefficients component. A robustness check is reported for the conventional linear identity. In (Appendix A), the derivation of these bounds, such as the binary-outcome bounds, the mirror-property proof, and the nonlinear decomposition are provided.

There are two methodological decisions that warrant a quick explanation. The Fairlie decomposition of the income gap was not preferred, as it is based on sequential random matching of observations between groups and thus suffers from the sensitivity of the random draw and order of variables, which leads to high variance across many replications, while the nonlinear Oaxaca–Blinder decomposition of Yun, and of Bauer and Sinning, approach yields an exact and path-independent allocation of the explained component using a single pooled coefficient vector. Given that our covariate set contains several correlated service-use variables, path dependence would have been a material concern. The Wagstaff–van Doorslaer identity assumes a linear additive decomposition of a covariance, and the elasticity terms it requires are valid only for a model where the outcome is a linear function of the determinants, which is why we use a linear probability model for the concentration-index decomposition. This is the conventional approach in health-inequality studies; and below we check that the linear probability estimates are consistent with the average marginal effects of the respective logistic models.

## 3 Results

### 3.1 Sample characteristics and summary statistics

The properties of the samples and the weighted summary statistics are given in Table 2. The weighted caesarean rate was 43.3%. Three-quarters (75.1%) were attended by a lady doctor, half (51.7%) in private facilities and 31.8% in public facilities and 14.0% at home. One-third of women (33.2%) had no formal education while 19.0% had higher education; 61.9% resided in rural areas. The mean age of the mothers was 29.5 years (SD 6.1) and the mean pregnancy order 3.3 (SD 2.0). There was a steady increase in caesarean section rate as wealth increased (from 21.0% in Q1 to 64.6% in Q5, *p* < 0.001), and as education level increased (from 29.4% among women with no education to 61.2% among those with higher education, *p* < 0.001). The rates were markedly different according to place of delivery (62.5% in private facilities, 32.0% in public facilities, and 0.1% at home; *p* < 0.001) and attendance (55.9% attended by a lady doctor, 5.1% attended by others; *p* < 0.001). As pregnancy order increased, so did the rate, dropping from 52.9% for first gestation, to 30.1% for order six or higher (*p* < 0.001), and the age gradient was small. These raw gradients are easily interpretable economically. The monotone quintile profile suggests that the surgical delivery demand is highly inelastic with respect to income for the relevant level of income. The close parallels between the education gradient and the wealth gradient are predicted by the Grossman model, which says that education increases efficiency in health production, as well as permanent income (10). The very low rates of C-section delivery at home and by community workers is a supply-side corner solution because the provision of surgical skills is absent from the community and at home, the ratio of the poor to C-section mechanically caps the rate of C-section. This strong negative pregnancy-order gradient is comparable to the world trend of increasing primary elective caesarean delivery rates for younger and lower-order pregnancies (6).

**Table 2:** Weighted summary statistics and caesarean section rates by background characteristics (*n* = 6,832).

Characteristic	Category	*n*	Weighted %	Caesarean % (weighted)	*p*-value
Wealth quintile	Q1 (poorest)	1710	20.0	21.0	<0.001
	Q2	1,434	20.0	36.5	
	Q3	1,300	20.0	44.6	
	Q4	1,288	20.1	49.5	
	Q5 (richest)	1,100	19.9	64.6	
Place of delivery	Home	1,061	13.9	0.1	<0.001
	Public facility	2,240	31.8	32.0	
	Private facility	3,336	51.6	62.5	
	Community workers	79	1.1	1.3	
	Others	116	1.5	52.9	
Birth attendant	Other attendant	1868	24.9	5.1	<0.001
	Lady doctor	4,964	75.1	55.9	
Women’s education	None	2,605	33.2	29.4	<0.001
	Primary	1,362	19.4	41.0	
	Middle	721	11.3	43.2	
	Secondary	1,010	17.2	52.8	
	Higher	1,134	19.0	61.2	
Region	Rural	4,706	62.0	40.1	<0.001
	Urban	2,126	38.0	48.5	
Maternal age (years)	15–19	212	2.7	42.0	0.105
	20–24	1,345	19.5	42.2	
	25–29	2084	31.3	42.4	
	30–34	1700	25.2	46.9	
	35–49	1,491	21.3	41.2	
Pregnancy order	1	1,333	20.0	52.9	<0.001
	2–3	2,800	40.9	45.9	
	4–5	1754	26.1	38.3	
	6+	945	13.1	30.1	

Weighted with survey weights. *p*-values from design-adjusted Wald tests (weighted logistic regression with PSU-clustered standard errors). ‘Others’ comprises deliveries attended by nutrition assistants, vaccinators, traditional practitioners (hakeem/homeopath), pharmacies and unclassified providers; its elevated CS rate (52.9%, *n* = 116) most plausibly reflects unclassified private providers and possible misreporting, and results are unchanged when this category is merged with ‘private facility’. Kish effective sample size = 3,759 (weight design effect 1.82). Percentages may not sum to 100 owing to rounding.

### 3.2 Correlation analysis

The lady-doctor attendance, private-facility delivery, wealth index and education were positively and significantly correlated with caesarean delivery (Table 3), while the pregnancy order showed a negative correlation (Table 3). The education level shows strong correlation with wealth (ρ = 0.650), and moderate with the use of private facilities (ρ = 0.32) and attending the lady-doctor for services (ρ = 0.34), but not with urban residence. Since the magnitude of the pairwise correlation between all regressors is below 0.65, there are no major multicollinearity issues. The ordering is a hallmark of a mediated association, which is quantified by the decompositions below, with service use variables correlating more strongly with the outcome than wealth itself and wealth correlating with service use.

**Table 3:** Spearman rank correlation matrix.

Variable	1	2	3	4	5	6	7	8
1. C-section	1							
2. Wealth index	0.310***	1						
3. Private facility	0.437***	0.319***	1					
4. Lady doctor	0.452***	0.338***	0.391***	1				
5. Education	0.246***	0.650***	0.221***	0.272***	1			
6. Urban	0.094***	0.329***	0.087***	0.120***	0.275***	1		
7. Maternal age	−0.004	−0.035**	−0.037**	−0.037**	−0.056***	0.034**	1	
8. Pregnancy order	−0.146***	−0.252***	−0.131***	−0.165***	−0.255***	−0.058***	0.583***	1

*p < 0.05; **p < 0.01; ***p < 0.001. Education treated as ordinal (0 none – 4 higher).

### 3.3 Adjusted correlates of caesarean delivery: hierarchical models

The results of hierarchical logistic regressions are shown in Table 4. The reduced-form Model 1 (distal determinants only) shows a steep wealth gradient (OR 4.76, *p* < 0.001) and an independent association between higher education and caesarean delivery (OR 1.58, *p* < 0.001). The inclusion of the proximate service-use variables in Model 2 reduces the direct wealth association by half (Q5 OR 2.23, 95% CI 1.62–3.06) and abolishes the education association; the delivery by private facilities is the largest association (OR 3.26, 2.68–3.97) and the attendance of a lady doctor is the greatest single association (OR 8.58, 6.19–11.89). The antenatal care and complications scores are added to the model and these are both positively associated with having a caesarean delivery (OR 1.31, *p* = 0.001; OR 1.82, *p* < 0.001 respectively), but the wealth and service-use scores need to be very similar to the main models. The main structural result is the association Model 1 → 2: about half of the distal association is mediated by who delivers the delivery care, and the rest of the association is direct and continues within the delivery care categories. The Model 2–3 service-use coefficients are partly defined by the presence of a physician and are usually not delivered outside of a setting; hence, these service-use coefficients should not be interpreted as causal relationships. A sensitivity analysis with only facility births (n = 5,576) yields very similar estimates (private facility OR = 3.26; lady doctor OR = 8.21; Q5 OR = 2.26). Maternal age (OR 1.03 per year) and pregnancy order (OR 0.92) were both associated with caesarean odds. The urban coefficient was slightly negative conditional on the other covariates (OR 0.82, *p* = 0.033); however, this conditional coefficient may be due in part to suppression dynamics once the service-use variables are conditioned on, so we do not interpret the coefficient here (in the reduced-form Model 1 the coefficient is also close to unity).

**Table 4:** Weighted logistic regression: adjusted odds ratios for caesarean delivery under hierarchical specifications (*n* = 6,832).

Variable	Model 1: Reduced form	Model 2: + Service use	Model 3: + Clinical
Wealth Q2	1.99*** (1.60–2.47)	1.58*** (1.24–2.02)	1.52*** (1.19–1.94)
Wealth Q3	2.55*** (2.05–3.18)	1.60*** (1.25–2.05)	1.51** (1.18–1.93)
Wealth Q4	2.78*** (2.16–3.57)	1.74*** (1.31–2.31)	1.62*** (1.22–2.16)
Wealth Q5 (richest)	4.76*** (3.59–6.32)	2.23*** (1.62–3.08)	2.07*** (1.50–2.85)
Education: Primary	1.22* (1.01–1.48)	1.26* (1.01–1.56)	1.22 (0.98–1.52)
Education: Middle	1.13 (0.87–1.46)	1.03 (0.75–1.40)	0.99 (0.73–1.35)
Education: Secondary	1.39* (1.08–1.80)	1.32 (0.98–1.78)	1.24 (0.92–1.69)
Education: Higher	1.58*** (1.22–2.03)	1.38* (1.01–1.89)	1.30 (0.95–1.79)
Private facility (vs public/other)	—	3.51*** (2.91–4.24)	3.50*** (2.90–4.23)
Lady-doctor attendant	—	13.51*** (9.94–18.34)	13.10*** (9.65–17.80)
ANC 4 + visits	—	—	1.31*** (1.12–1.54)
Pregnancy complications	—	—	1.82*** (1.37–2.43)
Urban residence	0.88 (0.74–1.04)	0.80* (0.66–0.97)	0.79* (0.65–0.95)
Maternal age (years)	1.02*** (1.01–1.04)	1.02** (1.01–1.04)	1.02** (1.01–1.04)
Pregnancy order	0.89*** (0.84–0.94)	0.92** (0.87–0.98)	0.92** (0.87–0.97)

Odds ratios with 95% confidence intervals; *p < 0.05, **p < 0.01, ***p < 0.001. Reference categories: wealth Q1, no education, non-private place of delivery, non-lady-doctor attendant, rural. Model 2 collapses place of delivery to private vs other for comparability with the decompositions; home and community-worker deliveries contain one caesarean event each, and the five-category specification (with its unstable ORs for those cells) is available on request. PSU-clustered standard errors.

Models 2 and 3 are subject to an important interpretive note. The socioeconomic position-caesarean delivery relationship is mediated through private-facility delivery and lady-doctor attendance: an increase in socioeconomic position increases the likelihood of women reaching private, doctor-led care, and delivery in private, doctor-led care increases the likelihood of caesarean delivery. These are therefore mediators and not confounders of the relationship between wealth and caesarean delivery and conditioning on them is over-adjustment to estimate the overall relationship between wealth and caesarean delivery. The decrease in the wealth coefficient from Model 1 (Q5 OR 4.76) to Model 2 (Q5 OR 2.23) should thus be interpreted as a measure of the magnitude of the wealth gradient that works through the service-use pathway. Models 2 and 3 are decompositions of the total association, in terms of a direct component and a component transmitted through service use, respectively. Odds ratios adjusted for the variables of the services used themselves should not be interpreted as the causal effect of the services used as these are co-determined with the caesarean decision (Table 5).

**Table 5:** Multicollinearity diagnostics: variance inflation factors (VIF).

Variable	Model 1	Model 2	Model 3
Wealth Q2	1.55	1.57	1.58
Wealth Q3	1.73	1.78	1.79
Wealth Q4	1.94	2.02	2.05
Wealth Q5	2.27	2.40	2.45
Education: Primary	1.36	1.36	1.36
Education: Middle	1.32	1.32	1.33
Education: Secondary	1.57	1.57	1.60
Education: Higher	1.92	1.93	1.96
Private facility	—	1.24	1.24
Lady-doctor attendant	—	1.27	1.29
ANC 4 + visits	—	—	1.22
Pregnancy complications	—	—	1.02
Urban residence	1.14	1.14	1.14
Maternal age	1.58	1.59	1.59
Pregnancy order	1.71	1.73	1.73
Maximum VIF	2.27	2.40	2.45

All VIF values fall far below the conventional thresholds of 5 and 10, indicating no material multicollinearity. The highest values attach to the wealth-quintile indicators, as expected for a set of mutually exclusive dummy variables.

The variance inflation factors are good indicators of well-conditioned hierarchical specifications, none exceeding 2.45, well below 5 or 10, and highest for the wealth-quintile indicators as would be expected for a mutually exclusive set of dummies. So there has been no issue of Multicollinearity. The addition of the proximate service-use variables leads to significantly improved model performance: discrimination increases from AUC 0.689 in Model 1 to 0.820 in Model 2 to 0.824 in Model 3; the McFadden pseudo-R^2^ goes from 0.074 to 0.248 and 0.254; the Brier score goes from 0.216 to 0.167 and 0.166; and AIC goes from 8,677 to 7,053 and 7,006 (Table 6). Calibration slopes are all close to unity (1.03) suggesting no overall over- or under-prediction of risk by any of the three models. The Hosmer–Lemeshow test is statistically significant in Models 2 and 3 (*p* = 0.001 and *p* = 0.015, respectively), and the test is commonly known to be able to reject when there are very small deviations from perfect fit, so it should be interpreted with caution, and the decile-level agreement together with the near-unity calibration slopes indicate that calibration is satisfactory for the purposes of this analysis. There are no observations that are unduly influential, as shown from the influence diagnostics: the largest Cook’s distance is 0.0019, well below the standard value of 1, and 149 observations (2.18%) lie above the heuristic value of 4/n, with no individual observation being particularly influential.

**Table 6:** Model performance, calibration and influence diagnostics.

Model	AUC	McFadden R^2^	Brier score	Hosmer–Lemeshow	Calibration slope	AIC
Model 1	0.689	0.074	0.216	15.81 (df 8), *p* = 0.045	1.03	8,677
Model 2	0.820	0.248	0.167	25.61 (df 8), *p* = 0.001	1.03	7,053
Model 3	0.824	0.254	0.166	18.95 (df 8), *p* = 0.015	1.03	7,006

AUC = area under the receiver operating characteristic curve. Discrimination is acceptable in Model 1 and good in Models 2–3. Calibration slopes close to 1.0 indicate well-calibrated predicted risk. The Hosmer–Lemeshow test rejects in Models 2–3, a known property of the test at large sample sizes. Maximum Cook’s distance was 0.0019 (cut-off 1.0), indicating no unduly influential observations.

### 3.4 Wealth-related inequality: concentration curves and indices

The concentration curve of caesarean delivery is entirely below the line of equality (Figure 1) and equality is rejected at every one of 19 evenly spaced ordinates with PSU-cluster bootstrap standard errors and Bonferroni correction. The curves for delivery at private facilities and attendance by a lady doctor show the same pro-rich dominance. Each of the three concentration indices (0.195, SE 0.012; 0.344, SE 0.021; 0.337, SE 0.021) is significantly different from zero at z ≈ 16 (Table 7) and all agree that there is inequality in caesarean delivery. Private-facility delivery (CI = 0.157) and lady-doctor attendance (CI = 0.115) are also significantly pro-rich in themselves. All quintiles are above the upper limit of the WHO reference range, which is 10–15% (5), as shown in Figure 2. The larger Erreygers value (0.337) is because it accounts for the high mean of the outcomes and is not an indication of more inequality; Punjab’s standard value (0.195) lies within, but toward the upper end of, the range for most published estimates of the delivery-care indicators (15, 16, 35). The poorest 40% of women have about a quarter of all caesareans, whereas the richest 40% have well over half. More importantly, Figure 2 reveals that this is not scarcity-based inequality, but excess-based inequality; that is, even the poorest quintile is above the WHO reference band, and more caesareans are medically unnecessary, a configuration in which the equity and efficiency problems point to the same private, physician-led margin (5, 6, 8).

**Figure 1:**
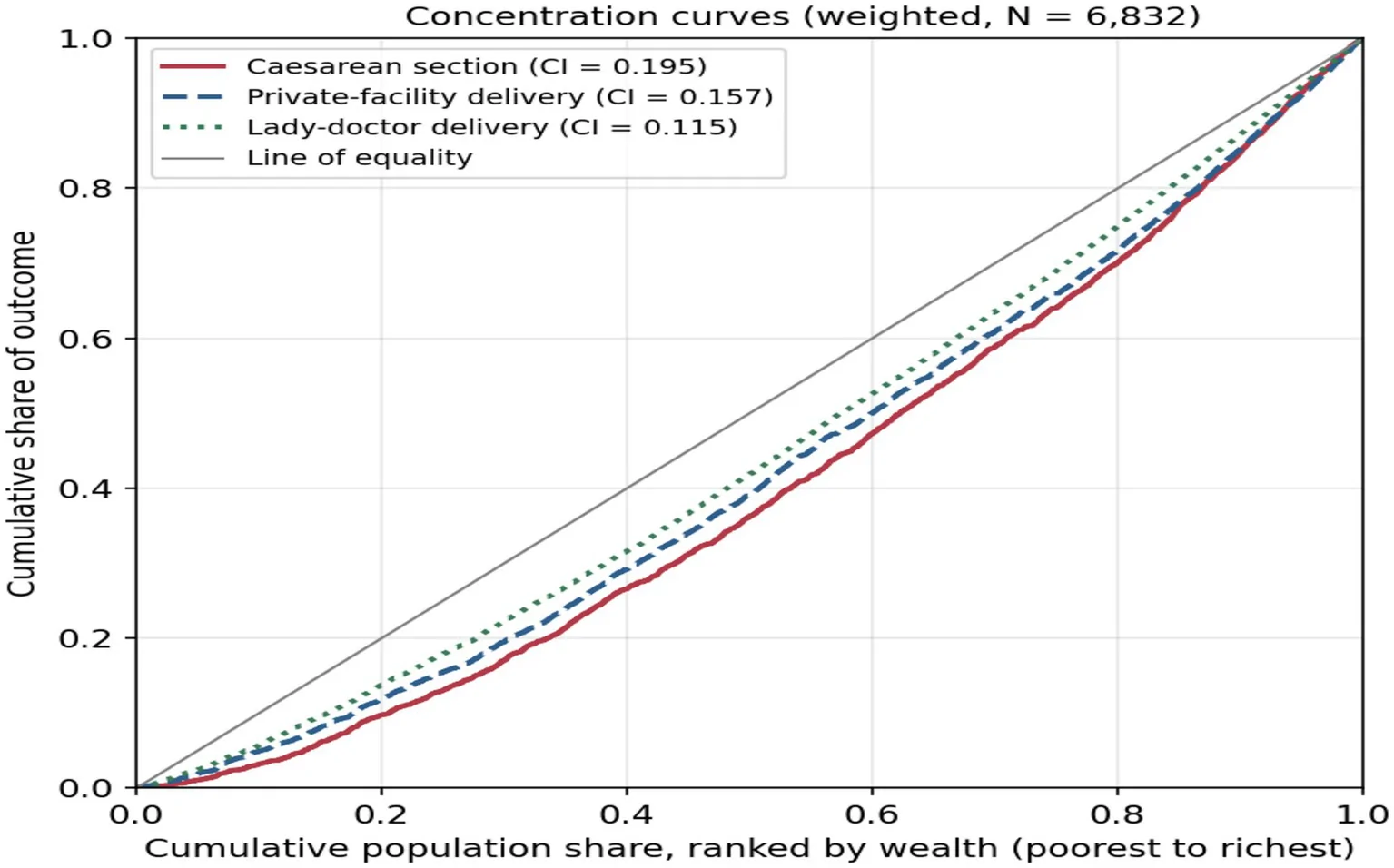
Concentration curves for caesarean section, private-facility delivery and lady-doctor attendance. All 19 tested ordinates of each curve lie significantly below the line of equality (cluster bootstrap, Bonferroni-corrected *p* < 0.05).

**Table 7:** Concentration indices (women ranked by PCA wealth score).

Outcome	Index	Estimate	SE	z	95% CI
C-section	Standard CI	0.1949	0.0122	16.0	0.1731 to 0.2147
C-section	Wagstaff-normalised	0.3435	0.0209	16.4	0.3105 to 0.3850
C-section	Erreygers	0.3373	0.0207	16.3	0.3006 to 0.3728
Lady-doctor delivery	Standard CI	0.1147	0.0055	20.7	0.1032 to 0.1250
Private-facility delivery	Standard CI	0.1568	0.0115	13.7	0.1332 to 0.1782

Weighted; standard errors from 500 PSU-cluster bootstrap replications. Positive values indicate concentration among wealthier women. Robustness: the standard CI is 0.196 under an extended asset index adding internet access, and 0.189 when wealth ranks are computed from separate urban and rural PCAs, indicating the result is not an artefact of urban bias in the asset index.

**Figure 2:**
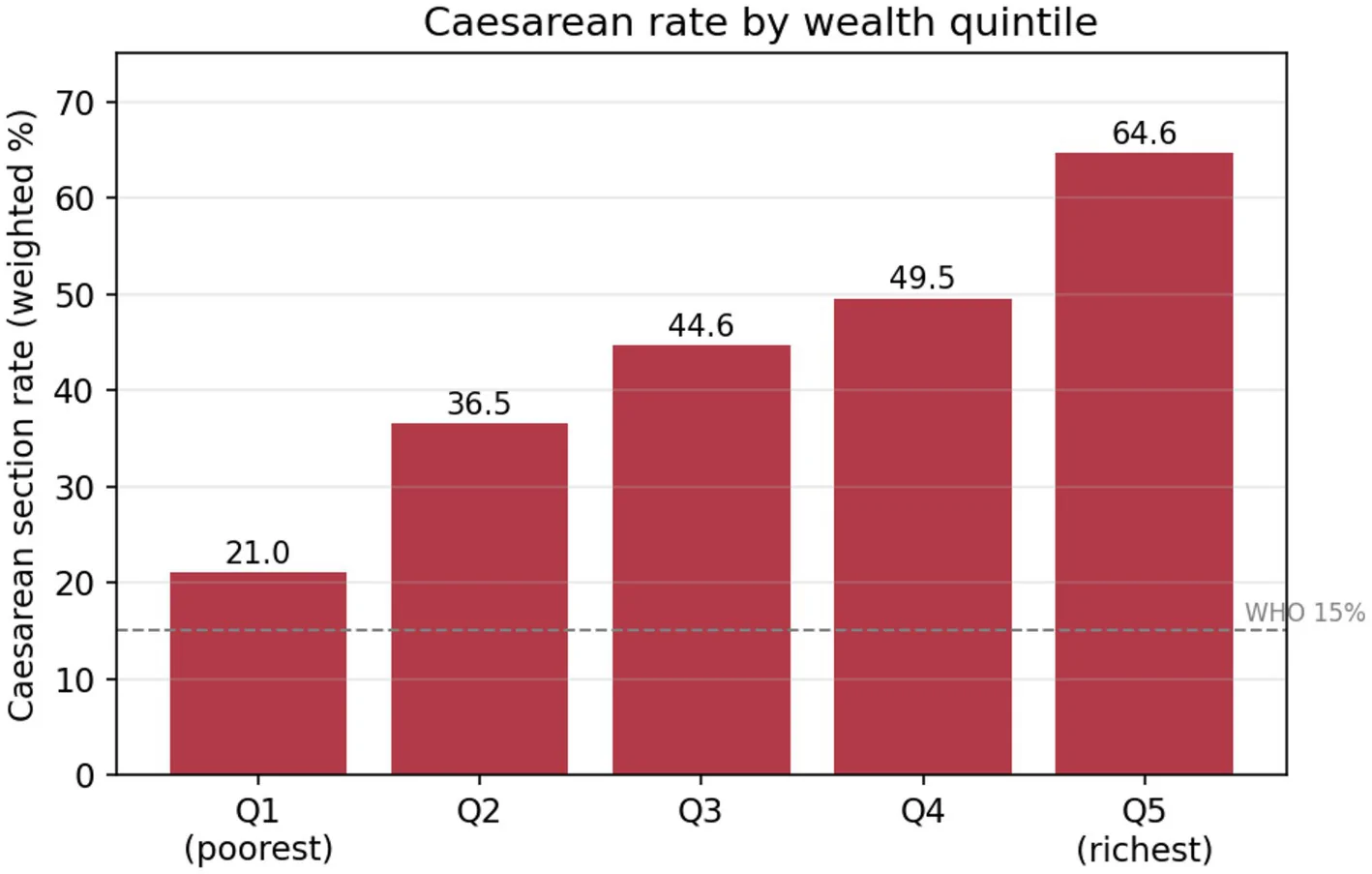
Weighted caesarean rate by wealth quintile; dashed line marks the 15% upper bound of the WHO reference range (5).

The 95% confidence intervals for the concentration indices, based on the percentile distribution of 500 PSU-cluster replications, are: standard CI 0.173 to 0.215; Wagstaff-normalised 0.311 to 0.385; Erreygers 0.301 to 0.373; lady-doctor delivery 0.103 to 0.125; and private-facility delivery 0.133 to 0.178 (Table 7).

### 3.5 Decision timing: elective versus emergency caesareans

Of those deliveries that were either recorded as a decision to do a caesarean section before labour started or were performed at the request of the mother, 79.4% were elected before labour started, increasing from 75.9% in the poorest quintile to 83.4% in the richest (Table 8). Pre-labour decision-making thus predominates in all levels of wealth and is likely to be more common with increasing wealth. This is more representative of scheduled primary caesareans, where provider discretion and convenience and economic considerations have the greatest leeway, as opposed to intra-partum emergencies as the primary proximate form of caesarean section delivery in Punjab.

**Table 8:** Timing of the caesarean decision by wealth quintile (caesarean deliveries only).

Wealth quintile	Elective (pre-labour) caesareans, % of CS (weighted)
Q1 (poorest)	75.9
Q2	74.3
Q3	77.0
Q4	81.4
Q5 (richest)	83.4
All quintiles	79.4

Weighted; item H04. Elective = decision made before the onset of labour; emergency = after onset. Timing was recorded for 2,772 caesarean deliveries with a small number of nonresponses excluded.

### 3.6 Decomposition of the concentration index

The main (Model 2) specification (Table 9; Figure 3) explains 98.6% of the measured concentration index, with the linear probability model producing a very close fit to the rank covariance, which is a measure of how well the model fits, not how complete. The largest single contribution is from lady-doctor attendance (36.1%) followed by private-facility delivery (24.4%). The direct contribution of the wealth quintiles is 29.6% and the direct contribution of education is 9.9%. This decomposition assigns 73.6% of the CI to wealth and 17.7% of the CI to education (95.9% accounted for overall) and, as can be seen in the comparison, about half of the contribution to the CI due to wealth in Model 1 is channelled through the two channels of service-use in Model 2. When the clinical covariates are added (Model 3) the picture remains the same (99.2% accounted for). There are two kinds of contributions: one is the attendance of lady doctors, which is very large in elasticity (ηk), and also very unequally distributed (Ck), so that it dominates; the other is education, which is very unequally distributed (Ck), but the conditional elasticity of this determinant is small when service use is held constant (ηkss).

**Table 9:** Wagstaff–van Doorslaer decomposition of the concentration index for caesarean delivery (Model 2).

Determinant	LPM coefficient	Mean	CI of determinant	Contribution	% of CI
Wealth Q2	0.0581	0.200	−0.4001	−0.0108	−5.5
Wealth Q3	0.0674	0.200	0.0002	0.0000	0.0
Wealth Q4	0.0801	0.201	0.4005	0.0149	7.6
Wealth Q5	0.1425	0.199	0.8006	0.0526	27.0
Primary education	0.0295	0.194	−0.1094	−0.0014	−0.7
Middle education	−0.0038	0.113	0.1046	−0.0001	−0.1
Secondary education	0.0462	0.172	0.3221	0.0059	3.0
Higher education	0.0619	0.190	0.5522	0.0150	7.7
Private-facility delivery	0.2545	0.516	0.1568	0.0477	24.4
Lady-doctor attendant	0.3530	0.751	0.1147	0.0703	36.1
Urban residence	−0.0421	0.380	0.2706	−0.0100	−5.1
Maternal age	0.0042	29.505	−0.0030	−0.0009	−0.4
Pregnancy order	−0.0134	3.299	−0.0893	0.0091	4.7

Based on a weighted linear probability model (LPM). Contribution of determinant k = (βk × mean k/mean caesarean rate) × CIk. Total CI = 0.1949; sum of contributions = 0.1922 (98.6%); residual = 0.0027. 13.3% of LPM fitted probabilities fall outside [0, 1]; LPM coefficients are consistent with average marginal effects from the logistic Model 2. The Wagstaff–van Doorslaer identity is defined for the linear model; the main Oaxaca–Blinder analysis is nonetheless conducted in nonlinear form (Table 10), and Table 11 shows the two agree closely.

**Figure 3:**
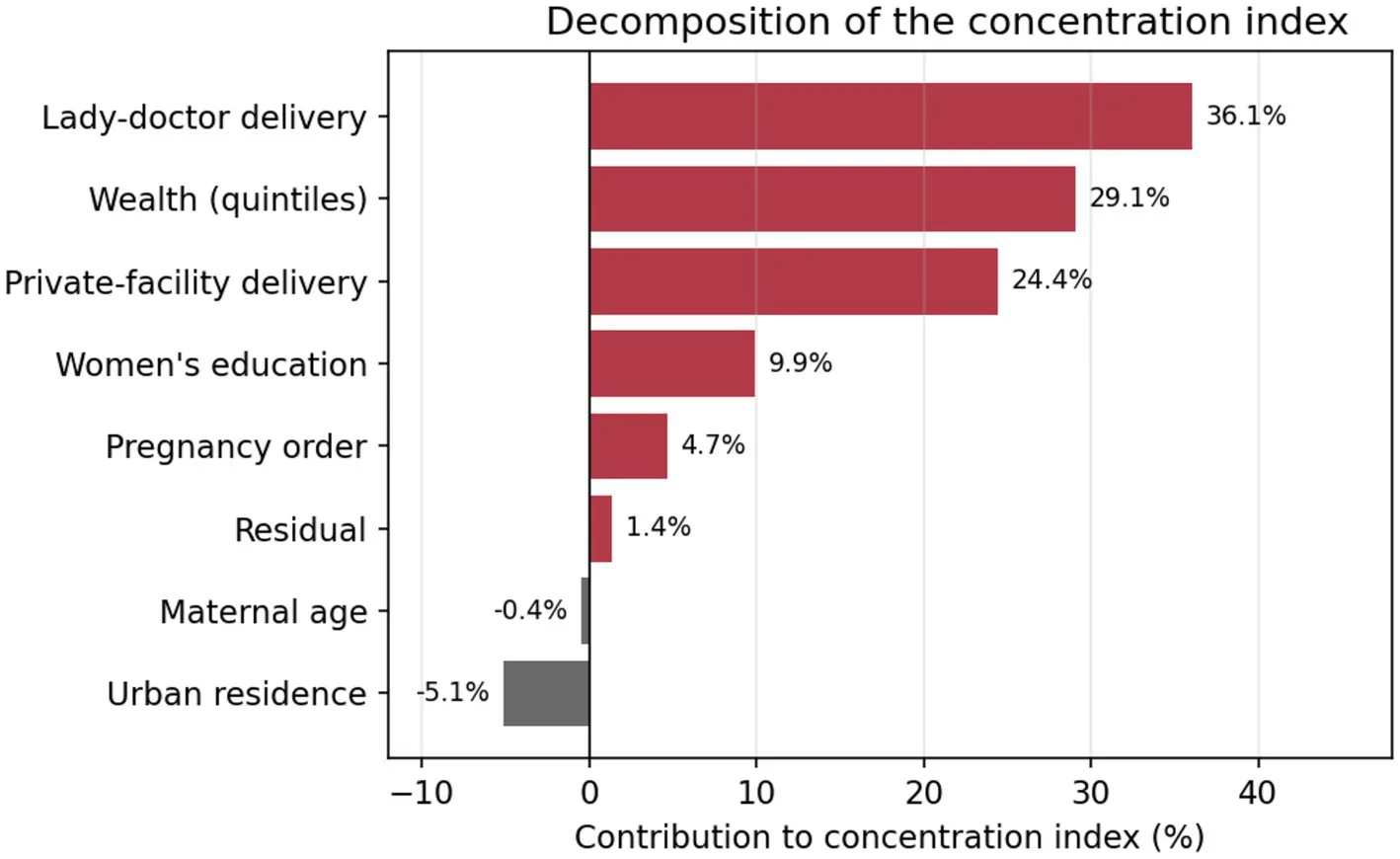
Grouped percentage contributions to the concentration index (Model 2).

### 3.7 Nonlinear Oaxaca–Blinder decomposition of the rich–poor gap

Since the dependent variable is binary, the two-fold decomposition of Yun (34), generalised by Bauer and Sinning (33), is the main decomposition applied: counterfactuals are averages of predicted probabilities from the pooled weighted logit and the explained component is distributed across determinants by Yun’s coefficient-weighted characteristics differences. The weighted caesarean rate was 57.1% among the richest 40% of women compared to 28.7% among the poorest 40% (28.4 percentage points difference) (Table 10; Figure 4). The differences in observed characteristics explain 24.8 points or 87.3% of the difference (cluster bootstrap SE 1.5 pp) and 3.6 points or 12.7% (SE 1.1 pp) remains unexplained. When the presence of a lady doctor and private facilities for delivery are taken into account, they account for 44.7 and 20.9%, respectively, of the difference; higher and secondary education together account for 20.1% and richer women’s lower pregnancy order accounts for 6.1%. Compared to the linear decomposition, the nonlinear approach assigns a greater proportion to the large coefficient of the lady-doctor attendance due to the fact that the logit takes that coefficient and applies it to the curvature of the probability function, instead of at a constant marginal effect. The sensitivity analysis is reported in Table 11. The accounted-for share is comparable for the link function (probit: 87.5%), the linear specification (87.0%) and group definitions (Q5 vs. Q1: 93.9% of a 43.6-point gap; median split: 85.4% of a 23.7-point gap). The share of the gap accounted for by the variables observed in the nonlinear reduced form is 66.9%, with the remainder of the gap (about 20 percentage points) attributable to the service-use variables in the proximate channel. If the characteristics of poor women could be observed and matched with those of the rich, the remaining 24.8 out of the 28.4 points (close to four times the WHO ceiling) would be associated with convergence. Raising the level of equity to the current level of behaviour of the richest would then exacerbate rather than solve the over-use issue — the arithmetic argument for pairing access extension for the poor with utilisation governance at the top of the distribution. The positive and precisely estimated 12.7% unexplained component is consistent with the prediction of differential returns: models of provider agency with remunerative clients predict that better experiences with the system happen to wealthier clients at the same observed characteristics (11–13).

**Table 10:** Detailed nonlinear (Yun) Oaxaca–Blinder decomposition of the caesarean gap, richest 40% vs. poorest 40%.

Determinant	Contribution to gap (pp)	% of gap
Primary education	−0.78	−2.8
Middle education	0.30	1.1
Secondary education	2.12	7.5
Higher education	3.60	12.7
Private-facility delivery	5.94	20.9
Lady-doctor attendant	12.69	44.7
Urban residence	−0.61	−2.2
Maternal age	−0.22	−0.8
Pregnancy order	1.74	6.1

Nonlinear two-fold decomposition (33, 34): counterfactuals are averaged predicted probabilities from the pooled weighted logit; detailed contributions use Yun’s weights. Gap = 28.4 pp; characteristics component = 24.8 pp (87.3%); coefficients component = 3.6 pp (12.7%). Cluster bootstrap standard errors (300 PSU replications): explained 1.5 pp, unexplained 1.1 pp.

**Figure 4:**
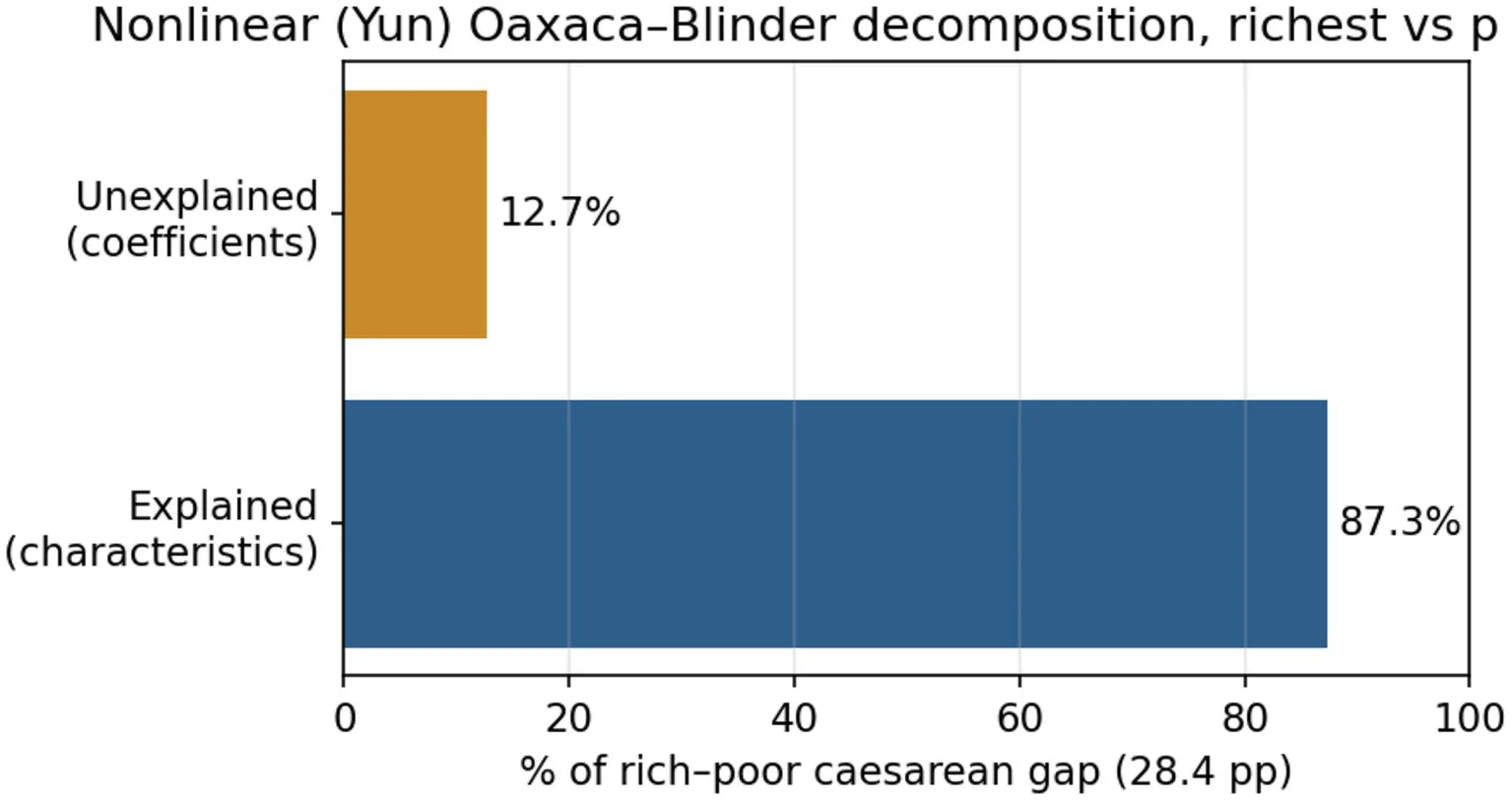
Aggregate nonlinear (Yun) Oaxaca–Blinder decomposition (main specification).

**Table 11:** Sensitivity of the decomposition results.

Analysis	Specification/contrast	Gap (pp)	Explained (%)
Oaxaca–Blinder (main)	Nonlinear logit-based (Yun), richest 40% vs. poorest 40%	28.4	87.3
Oaxaca–Blinder	Nonlinear probit-based, 40/40	28.4	87.5
Oaxaca–Blinder	Linear (LPM), pooled coefficients, 40/40	28.3	87.0
Oaxaca–Blinder	Nonlinear, Q5 vs. Q1	43.7	93.9
Oaxaca–Blinder	Nonlinear, top half vs. bottom half	23.7	85.4
Oaxaca–Blinder	Nonlinear, reduced form (service-use variables excluded)	28.4	66.9
WvD decomposition	Model 1 (reduced form): share of CI accounted for	—	95.9
WvD decomposition	Model 2 (+ service use)	—	98.6
WvD decomposition	Model 3 (+ clinical covariates)	—	99.2
Concentration index	Extended asset index (17 items + internet)	—	CI = 0.196
Concentration index	Region-specific (urban/rural) PCA, within-region ranks	—	CI = 0.189
Logistic regression	Facility births only (*n* = 5,576): private OR 3.26, lady doctor OR 8.21	—	—

The 40/40 contrast is the main specification because it balances group sizes while preserving a sharp wealth contrast; results are not sensitive to this choice. The reduced-form row excludes private-facility and lady-doctor variables from the covariate set. Concentration-index rows report the standard CI of caesarean delivery under alternative wealth-index constructions (baseline 0.195).

## 4 Discussion

The objective of this study was to measure and decompose the inequality of wealth in caesarean delivery in the largest province of Pakistan, Punjab, and four major findings are drawn. Caesarean delivery is very common in this sample: 43.3% of last deliveries; and is higher than the WHO 10–15% reference range in all wealth quintiles (including the poorest quintile, at 21.0%). Second, the inequality is considerable, with the standard concentration index at 0.195, which is in the upper half of the range seen with delivery-care indicators in low- and middle-income countries (15, 16, 35). Third, the hierarchical decompositions provide measures of composition of the inequality: the rich–poor gap is about two-thirds due to distal characteristics alone (66.9%, nonlinear decomposition) and the two proximate variables of service use together account for about three-fifths of measured inequality (87.3% accounted for overall). Roughly three-fifths of measured inequality is due to the combination of the two proximate service-use variables, with lady-doctor attendance accounting for about one-third and private-facility delivery accounting for the other. Fourth, the unexplained component of the gap is small but non-negligible (12.7%) and is estimated with precision, and is positive, suggesting that there are systematic differences in the likelihood of surgery among women with similar observed characteristics. The service-use variables are co-determined with the caesarean decision, in that surgery requires a physician and in practice a facility. The results fall comfortably into the literature on physician agency and supplier-induced demand in health economics. According to theory, and the evidence in the United States, caesarean intensity is sensitive to fee differentials and demand conditions (9, 11, 12), and better-informed patients are less likely to have a section (13). These conditions are compressed into the private obstetric market in Pakistan, for which there are fee-for-service payments, caesarean rates in multiples of the normal delivery rate, little use of utilisation review, and populations with an increasing ability to pay when clinical oversight is the least. In line with this, the results of the decision-timing analysis now reported formally in Table 8 — 79.4% of the caesareans were decided prior to labour, increasing from 75.9% in the poorest to 83.4% in the richest quintile — support this interpretation because there is evidence that this occurs in other comparable South Asian settings, particularly in the margins on which the convenience, scheduling economics and provider discretion operate most freely, as documented by Betrán et al. (36), McGuire (14), and Neuman et al. (25). They are undoubtedly on the demand-side as well: for the wealthy urban family the cost of long work is high, and a planned caesarean does not involve risk of time wastage — a time-price argument as familiar in the demand for medical services as it is in medical care (10). The positive unexplained component is consistent with supply-side behaviour acting on remunerative clients, but preference heterogeneity cannot be disentangled from provider inducement from these data. This outcome also provides some insight into the financing aspect of Pakistan’s UHC strategy. A caesarean is at the same time rationed by price for the poor and sold at a premium for the rich: this is the textbook scenario in which just one instrument cannot fix both failures (22). The second constraint is eased by the expansion of the Sehat social insurance programme to inpatient maternity care (23) and the decomposition results suggest that it should be equity-improving, because the accounted-for part of the gap is largely driven by the access variables that insurance seeks to address. Similarly, insurance that pays for caesarean delivery at a price premium, in the context of an un-audited private sector, runs the risk of financing the over-use margin and turning public subsidy into supplier rents (11, 12, 36). It is therefore not an optional refinement to have payment design that neutralises the caesarean–vaginal fee differential; that is the necessary condition for coverage expansion to benefit welfare. The comparison with the epidemiological literature reinforces it. Interventions shown to reduce unnecessary caesareans (mandatory second opinion, Robson-classification audit and feedback, midwife-led continuity of care) are interventions that work on provider behaviour and care organisation, and not on maternal demand (36–38). This is consistent with our findings that education had almost no conditional association with caesarean delivery after restricting to a sample of providers with similar rates of service use, although some conditional association was found in the reduced form. On the other side, the low rates of caesareans at home and by community workers are a measure of the remaining access challenge—when women have no access to facility care, the restricting factor is the availability and accessibility of comprehensive emergency obstetric services (2, 6). The failure is thus double in Punjab and the decompositions provided here are, to our knowledge, among the first quantitative ones in Pakistan of the gap between access-related and behavioural components of a provincial caesarean gap.

There is a qualification to be met on the WHO reference range. The 10–15% range is still accepted as an acceptable population level but the current WHO guidelines highlight that there is no universal optimal level of caesarean rates and that use should be guided by clinical need rather than a target. We therefore regard the reference range as a useful descriptive indicator which contextualizes the rate in the international context and do not infer from a rate exceeding the range that in every case the procedure might have been unnecessary. What our data do tell us is that caesarean use varies steeply by wealth, and does not relate to any clinical indicator, an equity finding that does not depend on any threshold.

### 4.1 Limitations

There are some restrictions to highlight. First, the survey does not include information on the mode of previous delivery: the strongest clinical predictor of caesarean delivery and likely to be associated with wealth due to earlier exposure to high-caesarean private care, the estimated wealth and parity associations will be confounded by repeat-section dynamics. Substantive results are not affected by available clinical covariates (antenatal care, pregnancy complications) but unobserved clinical need cannot be fully excluded. Secondly, delivery mode, timing of making the decision, and complications were self-reported and not verified with the delivery facility records and lastly the data are limited to Punjab only.

Second, more generally, the survey fails to detect any clinical indications to cesarean delivery other than self-reported pregnancy problems. Fetal distress, breech and malpresentation, placenta praevia, cephalopelvic disproportion, hypertensive disorders of pregnancy such as pre-eclampsia, multiple gestation and previous uterine interventions such as surgery are unmeasured. However, a number of these indications may also be socially patterned, or they may be correlated with differential antenatal surveillance; the observed wealth gradient may therefore be attributable to a genuine differential medical indication, rather than differential access to care. While we do not consider complete confounding by need to be a plausible explanation for the size of the gradient and monotonicity, the clinical need association estimates presented here should be interpreted as associations that accounts for any socially patterned variation in clinical need, rather than estimates of unnecessary surgery.

## 5 Conclusions and policy recommendations

The caesarean delivery rate is too high overall (43.3% of the last births) and concentrated among wealthier families (all wealth quintiles above the WHO reference range). The contribution of observable characteristics to the measured inequality is almost universal, and its structure is evident: having unequal access to attendance by a lady doctor or delivery in a private facility accounts for some three-fifths of the inequality; the direct purchasing-power gradient accounts for less than a third; and that of maternal education accounts for essentially none, once service use is held constant. The uncontrolled, but well quantified, additional component suggests, further, that the experiences of the richer clients are translated into surgery at a higher proportion. The caesarean problem in Punjab is thus not just a problem of knowledge or preference for mothers, but one of the structure of the delivery system, incentives and financing of the providers, and it needs to be tackled at both ends of the spectrum: at the bottom (residual unmet need) and at the top (discretionary over-use). The decomposition evidence leads to five policy directions. (1) Provider-payment reform: include an incentive that levels provider payment between caesarean and uncomplicated vaginal delivery (11, 12) in provider-payment budgets in health insurance and public-sector budgets. (2) Private sector clinical governance: mandate that elective caesarean indications be reported for Robson classification and that, as a condition of obtaining a licence for the facilities, and empanelment within the public insurance schemes, elective caesareans be subject to periodic audit, with particular attention paid to pre-labour primary sections, accounting for 79% of elective caesareans in these data (36, 37). (3) Specific financial safeguards: pool insurance for medically indicated caesarean section for the bottom two-fifths of earners, but not facilitate more generous benefit coverage for caesarean section than vaginal delivery. (4) Reinforce the non-surgical delivery channel: invest in midwife-led continuity of care and functional public comprehensive emergency obstetric facilities, the interventions most proven to reduce caesarean use safely without compromising outcomes (38). (5) Information and monitoring infrastructure: regularly report caesarean rates on a provincial level by facility ownership, Robson group and wealth proxy, which ensures that the equity and efficiency margins determined here are continually visible to regulators. Punjab’s challenge is to ensure that the expansion of surgical childbirth delivers financial protection to those in need, as the SDGs promise, while governing the private margin whose growth now produces more provider revenue than maternal health gain (1, 5, 6).

The findings are association based. Analysis of the study design and decomposition results show that the inequality measured is distributed as observed across the determinants of inequality, and do not imply causal influences; the policy directions presented should therefore be understood as priorities for further study and evaluation as evidence-informed causal remedies.

## Data Availability

The data can be made available upon reasonable request from corresponding author.
