# Supplementary material for: Inequality atop excess: a nonlinear decomposition of wealth-related inequality in caesarean section delivery in Punjab, Pakistan
Source: Front Public Health. 2026 Sep 30;14:1930664. doi: 10.3389/fpubh.2026.1930664 (PMC13661399; doi:10.3389/fpubh.2026.1930664)
Supplement: Supplementary file 1 [file Supplementary_file_1.docx]

# **Appendix A: Mathematical derivations of the applied techniques**

**A.1 Spearman rank correlation.** The Pearson correlation coefficient applied to the ranks is a Spearman coefficient. Ranks of n distinct values have mean R̄ = (n+1)/2 and variance Var(R) = (n²−1)/12. Substituting these moments into the Pearson formula ρ = cov(R(x),R(y)) / [σ_R(x) σ_R(y)] and writing dᵢ = R(xᵢ) − R(yᵢ), so that Σdᵢ² = ΣR(xᵢ)² + ΣR(yᵢ)² − 2ΣR(xᵢ)R(yᵢ), yields after simplification the computational form ρ_S = 1 − 6Σdᵢ² / [n(n²−1)]. The significance is evaluated using t = ρ_S√[(n−2)/(1−ρ_S²)] with n−2 degrees of freedom.

**A.2 Logistic regression.** Let y*ᵢ = xᵢ′β + εᵢ be a latent propensity, with yᵢ = 1 when y*ᵢ > 0. Then P(yᵢ = 1 | xᵢ) = Λ(xᵢ′β) = e^xᵢ′β^/(1 + e^xᵢ′β^) which is equivalent to ln[pᵢ/(1 − pᵢ)] = xᵢ′β. Exponentiated coefficients are adjusted odds ratios: for a one-unit increase in xⱼ, odds(xⱼ+1)/odds(xⱼ) = e^βⱼ^. Using the score equations, Σᵢwᵢ(yᵢ−Λ(xᵢ′β))xᵢ = 0, maximisation of the weighted log-likelihood ℓ(β) = Σᵢ wᵢ[yᵢ ln Λ(xᵢ′β) + (1−yᵢ) ln(1−Λ(xᵢ′β))] is achieved. Inference is based on the cluster-robust sandwich variance V̂(β̂) = A⁻¹(Σ_g s_g s_g′)A⁻¹, which is consistent under arbitrary within-cluster correlation, where the summed score s_g is from PSU g and the weighted information matrix A.

**A.3 Concentration curve and index.** Rank women by wealth score and let L(s) be the proportion of caesarean deliveries received by the poorest s. The concentration index is given by C = 1 − 2∫₀¹ L(s) ds, which is twice the area between L(s) and the 45° line. Using the cov_w(y, R) form of the covariance term, and normalizing the weights, integration by parts gives the form obtained here: C = (2/μ)·cov_w(y, R), where μ is the weighted outcome mean (33, 36). When the outcome is binary, there is a shrinking of the attainable range. The Wagstaff normalisation is simply dividing by the attainable maximum value, W = C/(1−μ) (33). The Erreygers correction E = 4μC is subject to the mirror condition: cov(1−y, R) = −cov(y, R), thus E(1−y) = 4(1−μ)C₁₋ᵧ = −4μC = −E(y) (32). For the decomposition, write y = α + Σₖβₖxₖ + ε and compute the weighted covariances with R: cov(y,R) = Σₖβₖcov(xₖ,R) + cov(ε,R). It can be seen that by multiplying with 2/μ and by x̄ₖ/x̄ₖ, it becomes C = Σₖ(βₖx̄ₖ/μ)Cₖ + GC_ε/μ, where Cₖ is the concentration index of determinant k, and ηₖ = βₖx̄ₖ/μ is the elasticity of determinant k (26). A determinant does not have an independent contribution to the pro-rich inequality other than when multiplied by its elasticity and its concentration index, and the residual represents inequality not explained by the linear model, GC_ε/μ.

**A.4 Oaxaca–Blinder decomposition: linear and nonlinear extension (Yun).** Linear case: separate estimation of the linear probability model for the richest group (r) and the poorest group (p) yields OLS planes that go through the group means, ȳ_g = x̄_g′β̂_g, and the difference is Δ = x̄_r′β̂_r − x̄_p′β̂_p. The two-fold decomposition is generalised to the nonlinear case by Bauer and Sinning (34): the first component is the characteristics component — the difference in averaged counterfactual probabilities evaluated at the same coefficients β* (here the pooled weighted logit) — and the second is the coefficients component. As F is nonlinear, the characteristics component is not additive across variables; Yun (35) resolves this by making a first-order approximation, which results in weights Wₖ = (x̄_rk − x̄_pk)β*ₖ / Σⱼ(x̄_rj − x̄_pj)β*ⱼ where Σ Wₖ = 1; the detailed contribution of determinant k is computed as Wₖ × characteristics component. The weights are exact in the linear model, and in the empirical application differ by less than half a percentage point of the gap from the linear and nonlinear aggregate results (Table 9). Inference is based on PSU-cluster bootstrap resampling.
